# Patients With SESA Syndrome May Not Have a Good Outcome: A Case Series

**DOI:** 10.1155/carm/8785833

**Published:** 2025-12-18

**Authors:** Jovana Ivanovic, Masa Kovacevic, Tamara Svabic Medjedovic, Vanja Radisic Vukomanovic, Branislav Ralic, Aleksandar Ristic, Dejana Jovanovic, Ivana Berisavac

**Affiliations:** ^1^ Neurology Clinic, University Clinical Center of Serbia, Belgrade, Serbia, kcs.ac.rs; ^2^ Faculty of Medicine, University of Belgrade, Belgrade, Serbia, bg.ac.rs; ^3^ Neurology Department, CHC Zvezdara, Belgrade, Serbia

**Keywords:** alcohol, encephalopathy, epilepsy, SESA

## Abstract

**Introduction:**

Subacute encephalopathy with seizures in alcoholics (SESA) is a rare complication of chronic alcohol abuse manifested as seizures, altered mental state, and focal neurological deficits. Electroencephalography usually shows focal epileptic anomalies, and brain MRI reveals reversible focal cortical or subcortical T2w/FLAIR hyperintense lesions. This syndrome is still underrecognized but a very important entity with a high risk of recurrent seizures requiring long‐term antiseizure polytherapy treatment and has an uncertain outcome.

**Case Series:**

We report four male patients who presented with focal motor seizures or status epilepticus associated with chronic alcohol intake. They were treated with antiseizure polytherapy combined with other intensive symptomatic therapies. Two patients had a good outcome without new seizure occurrences, while the other two patients, despite intensive care treatment, developed multiorgan dysfunction and had a fatal outcome.

**Conclusion:**

SESA is a life‐threatening disease with an uncertain outcome, especially if hospitalization in intensive care units is needed.

## 1. Introduction

Subacute encephalopathy with seizures in alcoholics (SESA) is a rare but underrated condition associated with chronic alcohol abuse [1]. The clinical presentation includes seizures, typically nonconvulsive status epilepticus (NCSE), impaired consciousness, focal neurological deficits, focal electroencephalographic discharges, including lateralized periodic discharges (LPDs), and reversible T2w/FLAIR hyperintense cortical lesions on brain MRI [2]. The SESA syndrome is associated with recurrent seizures, necessitating aggressive antiseizure medication (ASM) [3, 4]. We present a case series of four patients with SESA syndrome who were treated in the neurology intensive care unit (NICU).

## 2. Case Series

### 2.1. Patient 1

**Figure 1:**
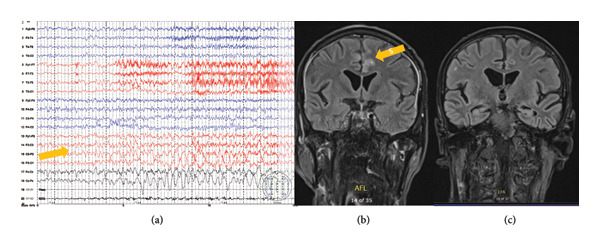
(a) Standard EEG confirmed subclinical focal seizures from the left centroparietal region, (b) brain MRI showed a T2w/FLAIR hyperintense lesion in the superior frontal and cingulate gyrus bilaterally, and (c) follow‐up brain MRI on T2w/FLAIR showed regression of previously identified hyperintense lesions.

Our first patient is a 62‐year‐old who was admitted to the NICU due to a series of focal motor seizures, without impaired consciousness, which were disrupted with diazepam and phenobarbital IV. His first epileptic seizure occurred 5 days before hospitalization. The patient was a chronic alcohol abuser who stopped using alcohol 3 days before the first seizure. On admission, he was hemodynamically stable, with normal vital signs. He was spatiotemporally disoriented, with postictal confusion. ASM was immediately started—levetiracetam (60 mg/kg of body weight) with high doses of Vitamin B1. Detailed diagnostic procedures including serological and cerebrospinal analyses which were all negative and excluded infectious, immune‐mediated, and neoplastic etiologies. Standard EEG (30 min of recording) showed three subclinical focal seizures from the left centroparietal region (Figure 1(a)), without criteria for NCSE. On the fourth day of hospitalization, the patient developed nonconvulsive focal status epilepticus which was treated with a loading dose of phenobarbital (20 mg/kg/h), without EEG improvement, and therefore, midazolam infusion was started (0.4 mg/kg/h), with serial EEG follow‐up. Also, the ASM was corrected with the initiation of valproic acid 2000 mg/day, pregabalin 600 mg/day, topiramate 600 mg/day, and phenobarbital 100 mg/day. Brain MRI showed a T2w/FLAIR hyperintense lesion, with restricted diffusion in the superior frontal and cingulate gyrus bilaterally, with cortical reductive changes, ischemic periventricular leukoencephalopathy, and chronic microischemic lesions (Figure 1(b)). Despite negative serological and CSF immunological analyses, having in mind new‐onset refractory status epilepticus (NORSE), unresponsive to the application of loading doses of various ASMs (diazepam, phenobarbital, levetiracetam, topiramate, pregabalin, and valproic acid), dependent on the application of anesthetic doses of drugs, we started immunotherapy as the patient met the criteria for NORSE. Therefore, we administered methylprednisolone 1000 mg/day intravenously for five consecutive days, with prednisone tapering, and performed five courses of therapeutic plasma exchange (TPE). A follow‐up brain MRI was performed 3 weeks after the disease onset and showed regression of previously identified hyperintense lesions (Figure 1(c)). The patient was seizure‐free and discharged on the 36th day of hospitalization.

### 2.2. Patient 2

**Figure 2:**
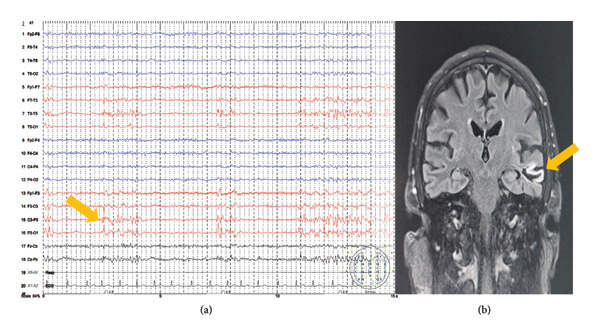
(a) Standard EEG with interictal activity on the left frontocentral region and LPDs at the same region and (b) endocranial MRI showed an extensive T2w/FLAIR hyperintensive lesion in the left temporal region.

The next patient is a 66‐year‐old alcohol abuser with a history of daily excessive drinking, who was hospitalized in the NICU with focal motor seizures evolving to bilateral tonic–clonic seizures aborted by propofol (100 mg intravenously) which was administered by an anesthesiologist, who first treated the patient in the resuscitation room. Levetiracetam therapy (60 mg/kg of body weight) was also started. On admission, the patient was hemodynamically stable but hypertensive with de novo atrial fibrillation, with serial fractures of the lumbar spine (L1–L3), and a dislocated XI rib on the right side. Neurological examination revealed mild sensory motor dysphasia and mild right hemiparesis. Brain CT showed chronic microischemic lesions in the basal ganglia bilaterally without acute lesions and normal other findings, while CT angiography of extracranial and intracranial blood vessels was normal. Serological and CSF analyses were normal, also performed per the same protocol. EEG showed 17 episodes of subclinical seizures with interictal activity on the left frontocentral region and LPDs at the same region (Figure 2(a)). The loading dose of phenobarbital was administered with ASM correction—levetiracetam (60 mg/kg of body weight) and topiramate 600 mg/day. Brain MRI showed an extensive T2w/FLAIR hyperintensive lesion in the left temporal region (Figure 2(b)), with restricted diffusion, and chronic microischemic lesions bilaterally in the basal ganglia, right thalamus, and right cerebellum. Cortical reductions were also confirmed. Seven days after the disease onset, the patient became hemodynamically unstable and developed respiratory insufficiency due to an acute myocardial infarction. He was intubated and connected to mechanical ventilation with continuous vasopressor therapy (norepinephrine), but despite all intensive treatment measures implemented, the patient passed away on the 20th day of hospitalization.

### 2.3. Patient 3

**Figure 3:**
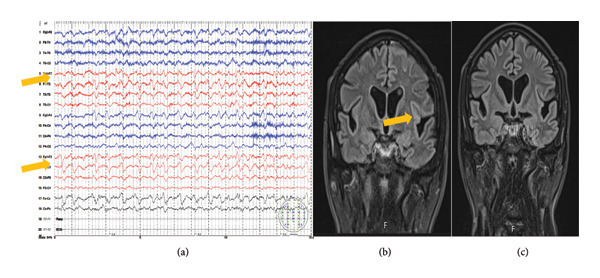
(a) Standard EEG showed ictal rhythm under the left FCT region, (b) brain MRI confirmed a T2w/FLAIR hyperintensive lesion in the left opercular‐insular region, and (c) follow‐up MRI with complete regression of previously verified lesion.

The third patient is a 55‐year‐old male, an active alcoholic abuser, who was admitted to the NICU due to multiple focal impaired awareness seizures and multiple fractures, terminated by a loading dose of phenobarbital. He was hemodynamically stable, eupneic on oxygen therapy, somnolent, without a focal neurological deficit. ASM was started—levetiracetam (60 mg/kg of body weight) and valproic acid (2000 mg/day). An extensive diagnostic protocol was also performed with negative results. Standard EEG showed ictal rhythm under the left FCT region (Figure 3(a)). The first brain MRI was performed on the fourth day of hospitalization and confirmed a T2w/FLAIR hyperintensive lesion in the left opercular–insular region and cortical reductive lesions with prominent perivascular spaces, indicating the presence of small vessel disease (Figure 3(b)). The cortical/subcortical lesion in the left hemisphere completely resolved on follow‐up MRI performed 6 days later (Figure 3(c)). The patient was discharged on the 13th day of hospitalization, without new epileptic seizures.

### 2.4. Patient 4

**Figure 4:**
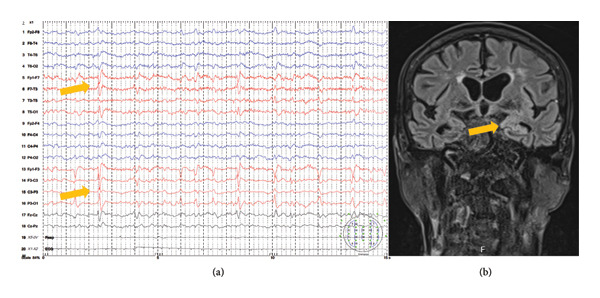
(a) Standard EEG revealed left arrhythmic LPDs and (b) endocranial MRI confirmed extensive T2w/FLAIR hyperintense lesions in the left thalamus, temporomesial, hippocampal, parahippocampal, insular, and cingulate gyrus.

Our fourth patient is a 66‐year‐old male suffering from compensated alcoholic liver cirrhosis and daily alcohol intake. He was admitted to the NICU with focal motor seizures evolving to bilateral tonic–clonic seizures and coma. Upon admission, he was intubated and placed on controlled mechanical ventilation, with normal other vital functions. He received treatment with 20 mg of intravenous diazepam followed by a loading dose of phenobarbital, which successfully aborted the status epilepticus. ASM polytherapy was prescribed, including levetiracetam 4000 mg/day combined with lacosamide 200 mg/day, and there were no further occurrences of seizures. Our diagnostic protocol excluded various etiologies. An EEG revealed left unilateral LPDs (Figure 4(a)), and a brain MRI showed extensive hyperintense lesions in the left thalamus, temporomesial, hippocampal, parahippocampal, insular, and cingulate gyrus, with restricted diffusion, without postcontrast enhancement, as well as cortical reductive changes, ischemic periventricular leukoencephalopathy, and chronic lacunar lesions (Figure 4(b)). The patient’s severe general condition persisted throughout the disease course, with the development of hemodynamic decompensation, sepsis, and multiorgan dysfunction, ultimately resulting in a fatal outcome on the 11th day of hospitalization.

## 3. Discussion

We presented four male patients with chronic alcohol abuse, clinical presentation manifested as impaired consciousness, prolonged focal motor seizures, and status epilepticus which required the administration of intensive ASM. Also, all patients had specific focal EEG discharges with subclinical focal seizures and LPD and cortical/subcortical T2w/FLAIR hyperintensive lesions, with restricted diffusion and low ADC, and with exclusion of other possible etiologies, the diagnosis of SESA syndrome was established in our group of patients. They were treated with ASM polytherapy combined with other intensive symptomatic therapies and had similar clinical, electrophysiological, and radiological findings suggestive of SESA syndrome.

SESA syndrome is a rare complication of chronic alcohol consumption, without an estimated incidence, and was first described in 1981 [1]. Typically, SESA syndrome manifests as encephalopathy combined with focal motor seizures evolving to bilateral tonic–clonic seizures, and also focal impaired awareness seizures, reversible focal neurological deficits, and specific EEG and brain MRI changes [5, 6]. Symptoms can occur during active alcohol intake or after the typical withdrawal interval [7]. All our patients suffered from chronic alcoholism. Three patients had daily alcohol abuse, but the first patient stopped drinking 3 days before admission. The pathophysiological mechanisms are not clearly understood, with possible influences of increased metabolic demand, hyperperfusion associated with ongoing seizures, and underlying brain dysfunction caused by chronic alcoholism [8]. These changes can lead to cytotoxic edema which is responsible for reversible MRI changes [2]. The clinical presentation includes an impaired mental state including lethargy, somnolence, and coma. Our patients also had impaired consciousness, presented as somnolence in three patients, while our last patient was comatose. Focal neurological deficits are also common in SESA syndrome and could be presented with various symptoms like hemiparesis and sensorimotor dysphasia [6], as also presented in our third patient who had mild right hemiparesis and dysphasia.

Epileptic seizures are a core clinical manifestation and usually include focal impaired awareness seizures and generalized tonic–clonic seizures (GTCs) [3], but NCSE can also be presented [5, 9]. In this group of patients, SESA syndrome can be misdiagnosed because NCSE can be confused with withdrawal syndrome. Patients from our NICU also had focal motor seizures, while two of them developed status epilepticus. A standard EEG is characterized by severe lateralized abnormalities including focal sharp waves, slowing or suppression of baseline activity, and LPDs [1, 10]. The possible explanation for LPD occurrence is focal neuronal hyperexcitability [10]. Our patients also had focal epileptic discharges on EEG, and 50% of them had unilateral LPD, both with fatal outcomes, and this is in concordance with previously published data [4].

SESA syndrome is also characterized by reversible MRI abnormalities presented as focal cortical T2w/FLAIR hyperintensive lesions with restricted diffusion and reduced ADC values, and leptomeningeal enhancement can also be observed [11]. Lesions are reversible, which is explained by local hyperperfusion induced by seizure, blood–brain barrier damage, and cytotoxic edema [11]. Our patients had typical cortical and subcortical lesions which were reversible in two patients on follow‐up imaging. Unfortunately, neuroimaging follow‐up was not performed in the second and fourth patients due to the lethal outcome of the disease.

These MRI lesions can mimic other diagnoses; therefore, we conducted extensive diagnostic procedures in all our patients to exclude infectious, neoplastic, and immune‐mediated etiologies for every patient, which ruled out other etiologies. Also, we performed a follow‐up brain MRI 3 weeks after the disease onset for the first patient which showed regression of previously identified hyperintense lesions (Figure 1(c)). Having in mind the clinical presentation and reversible MRI lesions, we also considered alternative diagnosis which can also lead to reversible MRI changes (posterior reversible encephalopathy syndrome and acute disseminated encephalomyelitis), but the clinical presentation and the absence of fulfilling the diagnostic criteria excluded these alternative diagnoses. Detailed analyses performed by the diagnostic protocol were all negative which also confirmed the diagnosis of probable SESA syndrome. On the other hand, intensive immunosuppressive therapy including high doses of methylprednisolone and TPE conduction was conducted in this patient, and a spinal tap was performed three times to rule out inflammatory CSF, which supported the diagnosis of probable SESA syndrome. Another possible etiology is seronegative autoimmune encephalitis, but this is a very rare condition, especially in chronic alcoholics.

Other typical neuroimaging findings such as cortical reductions are also presented in more than 60% of patients, as well as chronic microvascular ischemic changes [2]. Also, all our patients had cortical reductive changes, with the presence of chronic vascular macroangiopathic lesions and signs of small vessel disease. In addition, these acute lesions caused by cytotoxic edema are usually concordant with EEG discharges [3], as in our group of patients. Niedermeyer and authors reported a case series of patients with SESA syndrome and found that patients manifested as status epilepticus and prominent EEG abnormalities with focal features concordant with LPDs [1]. Radiological presentation can be very useful for diagnosis, as shown in the latest study, which also described cortical T2w/FLAIR hyperintensive lesions in frontal, temporal, parietal regions, cingulate cortex, or insula [4], but a thalamic lesion is also described as an atypical radiological presentation [5]. Our patients presented as status epilepticus also had characteristic MRI changes in some of these regions, followed by LPD pattern. It is also hypothesized that patients who develop SESA syndrome frequently have premorbid cerebral lesions, which in some situations such as alcohol withdrawal or metabolic dysfunction can become highly epileptogenic [12].

It is also very significant that during 2024, diagnostic criteria for SESA syndrome were proposed, and our patients mostly met [4]. In our patients, we considered NORSE and immune‐mediated etiology as the cause of symptoms, but the current proposed criteria could shorten the time to accurate diagnosis, thus avoiding the use of immunosuppressive therapy and its potential side effects. Our first patient met criteria for NORSE, but after the MRI findings, this diagnosis was excluded. Patients with NORSE sometimes have T2/FLAIR hyperintensive lesions in the mesial temporal lobe and rarely in the claustrum, which was not a case in our patient.

The case fatality rate among our patients was 50%. None of our patients had lethal outcomes caused by seizures and epileptic status but due to somatic complications and multiorgan dysfunction. Although the literature reports favorable outcomes in these patients, to the best of our knowledge, in the literature, there is only one reported patient with SESA syndrome who had a fatal outcome, which was caused by intercurrent pneumonia and somatic complications [4], as in our two patients. On the other hand, it is reported that up to 23% of patients with SESA require transfer to the ICU and 26% develop permanent neurological sequelae [4]. Therefore, we should carefully monitor these patients because they usually need treatment in the intensive care units, increasing the risk of developing complications of intensive care treatment.

The limitation of our case series is the small group of patients, as well as the missing MRI follow‐up in all the patients.

## 4. Conclusion

Epileptic seizures related to chronic alcoholism can be presented as SESA syndrome, which is still underrecognized but a very important entity with a high risk of recurrent seizures and an uncertain outcome. Although SESA syndrome is considered to have a good prognosis with chronic ASM treatment, our case series showed that patients who needed treatment in the NICU had a high risk of developing NICU‐related complications and consequent lethal outcome.

## Consent

Informed consent was obtained.

## Conflicts of Interest

The authors declare no conflicts of interest.

## Author Contributions

All authors have equal contributions.

## Funding

No funding was obtained for this study.

## Data Availability

Data sharing is not applicable to this article as no datasets were generated or analyzed during the current study.
